# Regulation Mechanism of Exogenous Brassinolide on Bulbil Formation and Development in *Pinellia ternata*

**DOI:** 10.3389/fpls.2021.809769

**Published:** 2022-01-05

**Authors:** Chenchen Guo, Jigang Li, Minghui Li, Xihang Xu, Ying Chen, Jianzhou Chu, Xiaoqin Yao

**Affiliations:** ^1^College of Life Sciences, Hebei University, Baoding, China; ^2^Institute of Life Sciences and Green Development, Hebei University, Baoding, China

**Keywords:** phytohormone, starch and sucrose metabolism, bulbil, chlorophyll fluorescence, photoprotection, brassinolide

## Abstract

The bulbil is the propagative organ of the *P. ternata*, which has a great effect on the yield of *P. ternata*. It is well known that plant hormones play important roles in bulbil formation and development. However, there is not clear about brassinolide (BR) regulation on bulbil formation and development. In this study, we revealed the effects of BR and BR biosynthesis inhibitors (propiconazole, Pcz) application on the histological observation, starch and sucrose metabolism, photosynthesis pathway, and hormone signaling pathway of *P. ternata*. The results showed that BR treatment reduced starch catabolism to maltodextrin and maltose in bulbil by decreasing BAM and ISA genes expression and increased cellulose catabolism to D-glucose in bulbil by enhancing edg and BGL genes expression. BR treatment enhanced the photosynthetic pigment content and potential maximum photosynthetic capacity and improved the photoprotection ability of *P. ternata* by increasing the dissipation of excess light energy to heat, thus reduced the photodamage in the PSII center. BR treatment increased the GA and BR content in bulbil of *P. ternata*, and decreased the ABA content in bulbil of *P. ternata*. Pcz treatment increased the level of GA, SL, ABA, and IAA in bulbil of *P. ternata*. BR regulated the signal transduction of BR, IAA, and ABA to regulate the formation and development of bulbil in *P. ternata*. These results provide molecular insight into BR regulation on bulbil formation and development.

## Introduction

*Pinellia ternata* is a perennial herb of the Araceae family and is widely used as a medicinal plant in Asia (Xue et al., 2019). The natural seed setting rate of *P. ternata* is very low, and it is mostly cultivated by asexual reproduction (Liu et al., 2012). The bulbil is the propagative organ of the *P. ternata*, thus the number of bulbils has a great effect on the yield of *P. ternata*. Luo et al. (2014) study showed that the bulbil primordia is the initial structure for the formation of bulbil in *P. ternata*, which is formed by the differentiation of thin-walled cells in the outer proximal part of the vascular bundle. The formation and development of bulbil is a complex process, which is regulated by a variety of factors, such as environment, phytohormone, and genetic (Wu et al., 2020). Previous studies showed that temperature affected the bulbil development of *Lilium longiflorum* (Kim et al., 2007). Polyploidization enhanced the number of bulbils in *P. ternata* (Yang P.P. et al., 2018). Gao et al. (2018) used transcriptome sequencing to analyze starch and sucrose metabolism during bulbil development in *Sagittaria sagittifolia* and identified a number of genes associated with starch and sucrose metabolism, suggesting that starch and sucrose metabolism is critical for formation and development of bulbil. In most plants, starch and sucrose are the main transport and storage forms of carbohydrates, respectively. Starch accumulation is a dynamic process, which is synthesized by storage organs (bulbil) from the cleavage products of sucrose, including the synthesis, transformation, transportation, and degradation of starch and sucrose (Zhang et al., 2017). Sucrose is essential for bulbil morphogenesis, while starch is essential for bulbil emergence and development in *Lilium davidii* (Li et al., 2014). Sucrose was the major form of soluble sugar during stolon formation in *Tulipa edulis* (Miao et al., 2016). Soluble sugars, especially sucrose, are inducers of underground storage organ formation (Fernie and Willmitzer, 2001).

It is well known that plant hormones play important roles in the formation and development of bulbil. Removing the flower bud of *Agave tequilana* promoted the formation of bulbil, while auxin (IAA) application at the cut surface of the flower bud inhibited the bulbil formation (Abraham Juarez et al., 2015). Navarro et al. (2015) study showed that cytokinin (CK) promoted the tuber formation of potato, while gibberellin (GA) and strigolactones (SL) inhibited tuberization. He et al. (2020) also reported that CK enhanced the production of bulbil in *Dioscorea zingiberensis*, and IAA regulated bulbil formation. However, there is not clear about the regulation of brassinolide (BR) on the formation and development of bulbil.

Brassinosteroids are a class of steroid plant hormones and are the “sixth class” of phytohormones after IAA, GA, CK, abscisic acid (ABA), and ethylene, and are widely distributed in plants, such as algae, mosses, and vascular plants (Chmur and Bajguz, 2021). BR is one of the first discovered of brassinosteroids, and is important in the regulation of cell division and expansion, stem elongation, root growth, shooting growth, xylem differentiation, etc. (Maia et al., 2018; Otie et al., 2021). BR improved leaf carbon assimilation rate by increasing photosynthetic capacity (Hu et al., 2013). BR not only regulates many physiological processes in plants, but also improved plant tolerance to abiotic stresses (Dehghan et al., 2020). Kaya et al. (2020) reported that 24-epibrassinolide improved strawberry tolerance to iron deficiency by up-regulating the ascorbate-glutathione cycle. 24-epibrassinolide alleviated the harmful effects of chromium toxicity in tomato plants (Jan et al., 2020). BR treatment improved pepper tolerance to water stress, which may be related to BR-induced NO production and NO-mediated antioxidant defense system (Kaya et al., 2019). Chen et al. (2021) showed that high temperatures reduced sugar utilization and spikelet formation of rice by decomposing BR. In addition, the BR regulation on plant growth and development was interrelated with other hormones, which formed a complex regulatory network regulating various physiological processes in plants (Gruszka, 2019).

There are two ways to analyze the effects of BR hormones on plants (Chmur and Bajguz, 2021). The first approach is to construct gene defects or overexpression of key enzymes in BR biosynthesis by mutation or gene-editing techniques. Ren et al. (2009) study showed that the psc1 mutation (a leaky mutation of dwf4) reduced BR biosynthesis and resulted in the growth suppression of *Arabidopsis*. The phenotypical overexpression of the ABS1 gene was similar to that of BR-deficient mutants in *Arabidopsis*, and exogenous application of BR was effective in rescuing the dwarf phenotype (Wang et al., 2012). The second and easiest method is to inhibit endogenous BR biosynthesis in plants by exogenous BR biosynthesis inhibitor. The effects of BR and brassinazole (BR biosynthetic inhibitor) treatment on protein, pigments, and monosaccharides of *Wolffia arrhiza* were analyzed (Chmur and Bajguz, 2021). Song et al. (2019) also reported the important roles of BR and propiconazole (BR biosynthesis inhibitor, Pcz) in the vegetative growth and reproductive development of soybean. There is still a lack of information about the effect of BR biosynthesis inhibitor on the formation and development of bulbil.

In this paper, we first divided the bulbil growing season into formation, differentiation, and expansion stages according to the bulbil morphology. The BR and Pcz treatments samples from the three stages were analyzed for histological observation, starch and sucrose metabolism, photosynthesis pathway, and hormone signaling pathway. Using the Illumina NovaSeq 6000 platform, we sequenced the transcriptome of bulbil samples at the expansion stage after BR and Pcz treatments. This work investigated the roles of BR in the bulbil formation process and provided comprehensive transcriptional information on bulbil formation and development of *P. ternata*.

## Materials and Methods

### Plant Material

This experiment was carried out at Hebei University. The seed bulbs of *P. ternata* were obtained from the herbal medicine planting base in Tianshui, Gansu province, China. The same size seed bulbs (diameter: 0.5–1.0 cm) were selected and then planted in plastic pot (37.5 cm in length, 27 cm in wide, and 27 cm in high) filled with humus soil. A total of 36 plastic pots were divided into three experimental groups with 12 plastic pots each, 24 plants per pot. After three-leaf fully expanded, the control group was sprayed with distilled water by manual sprayer every day, the BR group was sprayed with 0.1 mg/L BR by manual sprayer every day, and the Pcz group was sprayed with 1 μM Pcz by manual sprayer every day. Plant samples were collected during bulbil formation, differentiation, and expansion stages, and were rinsed off the soil with distilled water. Developmental stages were characterized by changes in bulbil morphology. The samples were immediately frozen in liquid nitrogen until further analysis.

### Measurements of Tuber and Bulbil Yield

Four biological replicates for each sample were randomly collected from forty individual plants. The tubers and bulbils were rinsed free of soil and were weighed.

### Histological Analysis

The samples containing about 1 cm of stem at the location of the bulbil were fixed in FAA fixation buffer (formaldehyde: glacial acetic acid: 50% ethanol = 1:1:18) for 48 h. The fixed samples were sequentially dehydrated in solutions of 30, 50, 70, 80, 90, 100, and 100% ethanol, followed by sequential immersion in solutions of xylene: ethanol 1:2, 1:1, 2:1, 100% xylene, and 100% xylene, and then the xylene in samples were gradually replaced with paraffin (melting point: 58–60°C) at 60°C. The sections of 8 μm thick were obtained using a rotary sectioning machine and were stained with 1% saffron T (w/v) and 0.1% fast green (w/v).

### Measurements of Starch and Soluble Sugar Content

The content of starch and soluble sugar was measured using the method of anthrone-sulfuric acid method (Liu et al., 2020). The 0.2 g fresh sample was extracted with 3 mL of 80% ethanol in a water bath (75°C) for 30 min and was centrifuged at 4,507 *g* for 5 min. The reaction solution included 0.1 mL supernatant, 0.9 mL 80% ethanol, and 5 mL 0.1% anthrone (m/v) and was incubated in a boiling water bath for 10 min. The absorbance of the reaction solution was recorded at 620 nm, and soluble sugar content was expressed as mg g^–1^ fresh weight (FW). After drying the residue, the 2 mL 2% hydrochloric acid was added to the test and was incubated in a boiling water bath for 1 h. The reaction mixture included 5 μL extract, 0.995 mL distilled water, and 5 mL 0.1% anthrone (m/v) and was incubated in a boiling water bath for 10 min. The absorbance of the solution was read at 620 nm. The starch content was expressed as mg g^–1^ FW.

### Measurements of Photosynthetic Pigments and Chlorophyll Fluorescence

Photosynthetic pigments content was determined according to the method described by Lichtenthaler and Wellburn (1983). Fresh leave (0.2 g) was extracted with 8 mL acetone (80%) in dark for 3 days. The absorbance of solution was measured at 470, 646, and 663 nm. The results were expressed as mg g^–1^ FW. The chlorophyll fluorescence parameters were measured using pulse-amplitude-modulation (PAM) fluorescence imaging system (IMG-K6, Walz, Germany). The middle leaf of each plant was selected. After 20 min of dark adaptation, the F_*V*_/F_*M*_ was measured under 0.8 s saturation pulse (8,000 μmol m^–2^ s^–1^). Then, the leaves were exposed to 186 μmol m^–2^ s^–1^ photosynthetically active radiation (PAR) to measure fluorescence kinetic curve. Subsequently, the photo-response curves were measured at different PAR.

### Measurements of Endogenous Hormone Content

The hormone content was using the method of enzyme-linked immuno sorbent assay. The 0.2 g fresh sample was extracted with 1.8 mL of phosphate buffer (PH 7.4) and was centrifuged at 440 *g* for 20 min. The IAA, GA, ABA, SL, CK, and BR assay kits from shanghai enzyme-linked biotechnology (China) were used. The specific operation steps were detailed in the kit instructions.

### RNA Isolation, cDNA Library Preparation, and Transcriptome Sequencing and Assembly

Total RNA samples were extracted using the omni-plant RNA kit (DNase I) (CoWin Biosciences, Beijing) following the provided protocol. RNA quality and quantity were assessed using a NanoDrop 2000, an Agilent2100 nano, and were checked by RNase-free agarose gel electrophoresis.

The libraries were prepared by Illumina TruSeq™ RNA sample prep kit technology. The complete mRNA was obtained by the enrichment of magnetic beads with oligo (dT), and then the mRNA was randomly broken by adding fragmentation buffer. The small fragments of 300 bp were screened out by the magnetic beads. First-strand cDNA was synthesized using random hexamer primer and reverse transcriptase, and then the second strand was synthesized. End repair mix was added to make the sticky end of the cDNA into a flat end, and then a “A” base was added to the 3′ end.

The libraries were sequenced on an Illumina NovaSeq 6000 platform according to manufactures’ instructions. After sequencing, software seqprep and sickle were used to remove the linker sequence, low quality reads, any anonymous nucleotides greater than 10%, and adapter to obtain clean data. The clean data was assembly using trinity software (Grabherr et al., 2011). The software transrate and busco were used to evaluate and optimize the assembly results. Finally, we obtained the unigenes database by aggregating the assembled sequences.

### Functional Annotation of Unigenes

The unigenes sequence of *P. ternata* bulbil was compared with the national center for biotechnology information non-redundant protein (NR), clusters of orthologous groups of proteins (COG), pfam, and swiss-prot database using diamond (*e*-value ≤ 1e-5) and hmmer3 software obtain a protein sequence highly similar to the unigenes sequence for functional annotation. The unigenes were also submitted to gene databases for annotation and biological pathway analysis by blast2go and kobas software comprising gene ontology (GO) and kyoto encyclopedia of genes and genomes (KEGG) database.

### Differentially Expressed Genes Analysis

DEseq2 (*p*-value < 0.05, | log2 (fold change) | ≥ 2) was used to evaluate DEGs among the control, BR-treated, and Pcz-treated sample as described by Love et al. (2014). Quantification of gene expression levels was based on transcripts per million reads (TPM) values. The GO and KEGG enrichment analysis of DEGs was prepared by goatools and the free online platform of majorbio i-sanger cloud platform. GO and KEGG terms with adjusted *p*-value (FDR) < 0.05 were considered significantly enrichment. Unigenes involved in starch and sucrose metabolism, photosynthesis pathway, and hormone signaling pathway were screened by searching the integrative annotation results, and heatmaps of these genes with different expression leaves in different treatments were generated.

### Verification of Gene Expression by qRT-PCR

qRT-PCR was performed to validate the 24 DEGs involved in the starch and sucrose metabolism, photosynthesis pathway, and hormone signaling pathway. The cDNA was synthesized by reverse transcription (HiScript III RT SuperMix for qPCR (+ gDNA wiper) using 1 μg mRNA as a template (Vazyme, Nanjing, China). The q-PCR mixture (20 μL per-volume) comprised of 10 μL ChamQ Universal SYBR qPCR master mix (Vazyme, China), 0.4 μL of each primer (10 μM), 1 μL of cDNA, and 8.2 μL of RNase-free water. The primers were designed according to the integrated DNA technologies and listed in Supplementary Table 1. Expression was normalized to the reference gene *P. ternata* 18S (Xue et al., 2019). The relative expression levels of genes were calculated based on the 2^–ΔΔCT^ method.

### Statistical Analysis

Statistical analyses of experimental data were performed using the software SPSS (26.0, IBM, Chicago, United States) and Excel 2016. Tukey’s test was used to analyze significant differences (*P* < 0.05). The results were expressed as the mean ± standard error, and the experimental data had four biological replicates.

## Results

### Morphological and Histological Observations on Bulbil Formation of *Pinellia ternata*

**FIGURE 1:**
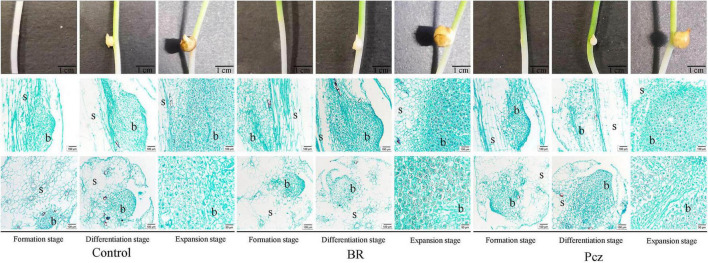
Anatomical and histological structure of *P. ternata* during bulbil formation and development. Scale bar for samples images = 1 cm; Scale bar for microscopy images = 100 or 50 μm. s, stem; b, bulbil.

The formation process of the bulbil was observed on the stem at formation, differentiation, and expansion stages (Figure 1). The early bulbil structure (white dot structure) was found in the formation stage, and a tight accumulation of cells was observed at this stage by histology. At the differentiation stage, the white-spotted structures bulged into white balls and differentiated to form the bulbil. The cells in the swelling region showed dense protoplasm. The number and size of cells were increased during the expansion stages, and black starch granules were observed in the cells. A higher number and size of starch granules were observed in the BR treatment, compared to the control, respectively.

### RNA-Seq Using Illumina Platform and Assembly of Unigenes in *Pinellia ternata*

The nine cDNA libraries of *P. ternata* bulbil were constructed from the high-quality RNA and were sequenced using the Illumina NovaSeq 6000 platform. These cDNA libraries generated clean reads of 57,117,606, 52,536,920, 53,506,752, 53,506,752, 50,190,552, 62,617,020, 55,852,426, 55,006,196, and 54,818,536, respectively. A total of 73.14 Gb clean data were produced, and the Q30 base percentage was at least 94.25%. Software trinity was used to *de novo* assemble all samples of clean data, and a total of 115, 445 unigenes with an average length of 754 bp and N50 equal to 1,189 bp were obtained. The length distribution of the *P. ternata* unigenes was shown in Supplementary Table 2. The fragment mapped percent of 48.72% was obtained by comparing these unigenes with the reference sequences assembled by trinity software. All these results indicated that the transcriptome sequencing data were reliable and can be used for further analyses.

### Function Annotation of Unigenes and Comparative Analysis of DEGs

The unigenes were annotated in the GO, KEGG, COG, NR, swiss-prot, and pfam databases. The 33,092 unigenes were annotated by the GO database. The classification of biological structure functions in the KEGG was assigned to metabolism, genetic information processing, environmental information processing, cellular process, organismal systems, and human diseases. In this way, 15,065 genes were annotated into the KEGG database. We found that 818, 697, and 707 unigenes were significantly differential expression in control vs. BR, control vs. Pcz, and BR vs. Pcz groups, respectively (Supplementary Figure 1). Subsequently, clustering analysis of differentially expressed genes was performed (Supplementary Figure 2). The GO and KEGG pathway enrichment analyses were conducted to further analyze the DEGs in BR and Pcz treatments.

### Effects of Brassinolide Treatments on Yield and Starch and Sucrose Metabolism in Bulbil of *Pinellia ternata*

**FIGURE 2:**
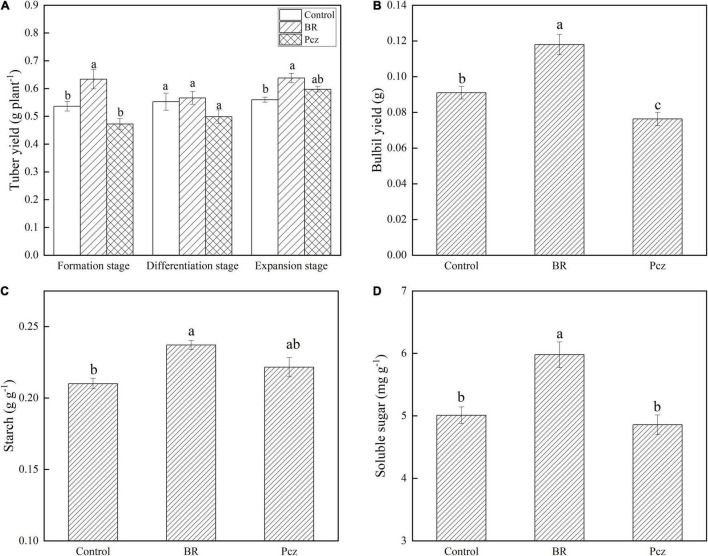
Tuber yield **(A)**, bulbil yield **(B)**, starch **(C)**, and soluble sugar **(D)** content in bulbil of *P. ternata* under BR and Pcz treatments. The bars with different letters are significantly different from each treatment (*p* < 0.05). Values are means of four replicates ± SE.

**FIGURE 3:**
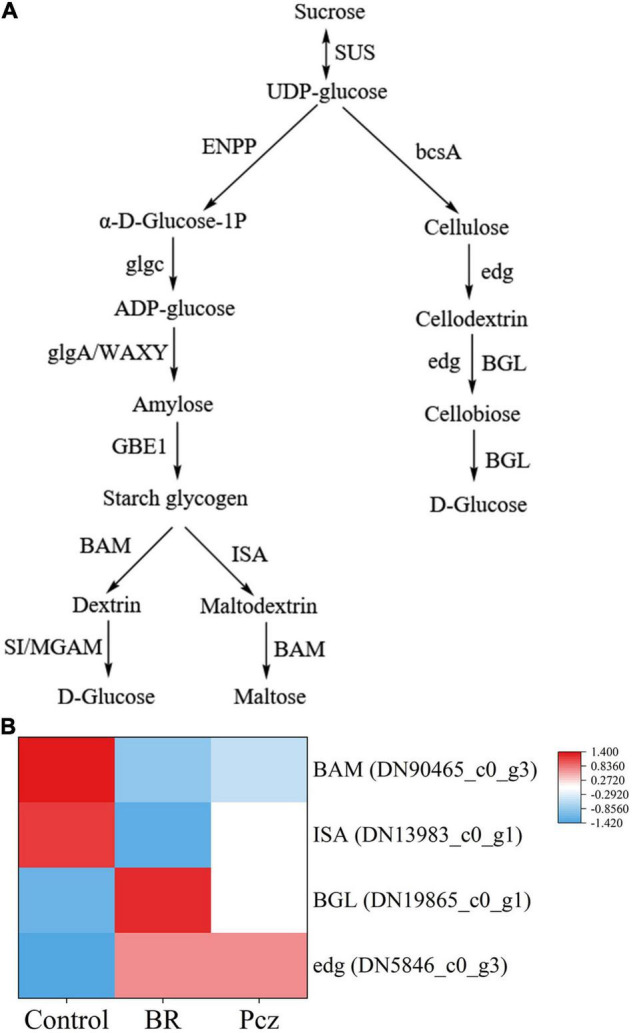
Analysis of differentially expressed genes related to starch and sucrose metabolism in bulbil of *P. ternata* under BR and Pcz treatments. **(A)** The starch and sucrose metabolism. **(B)** The expression levels of genes related to the starch and sucrose metabolism. Blue indicates a lower expression level, whereas red indicates a higher expression level. All data showed the average mean of three biological replicates.

The tuber yield was increased by 18.18 and 14.03% under BR treatment at formation and expansion stages, compared to the control, respectively (Figure 2A). The bulbil yield was increased by 29.67% under BR treatments and was decreased by 16.17% under Pcz treatments at the expansion stage, compared to the control, respectively (Figure 2B). The starch content in bulbil was increased by 12.87% under BR treatment at the expansion stage, compared to the control. Pcz treatments had no significant effect on the starch content in bulbil (Figure 2C). BR treatment increased the soluble sugar content by 19.32% in bulbil at the expansion stage compared to the control (Figure 2D). Heatmap analysis showed that the four DEGs involved in the starch and sucrose metabolism in bulbil of *P. ternata* (Figure 3). BR treatment significantly decreased the expression levels of beta-amylase (BAM) and isoamylase (ISA) genes, compared to the control, respectively. The expression levels of beta-glucosidase (BGL) and endoglucanase (edg) genes were significantly enhanced by BR treatment, compared to the control, respectively. The Pcz treatment had no significant effect on the expression levels of BAM, ISA, BGL, and edg genes, compared to the control, respectively.

### Effects of Brassinolide Treatment on Photosynthesis of *Pinellia ternata*

**FIGURE 4:**
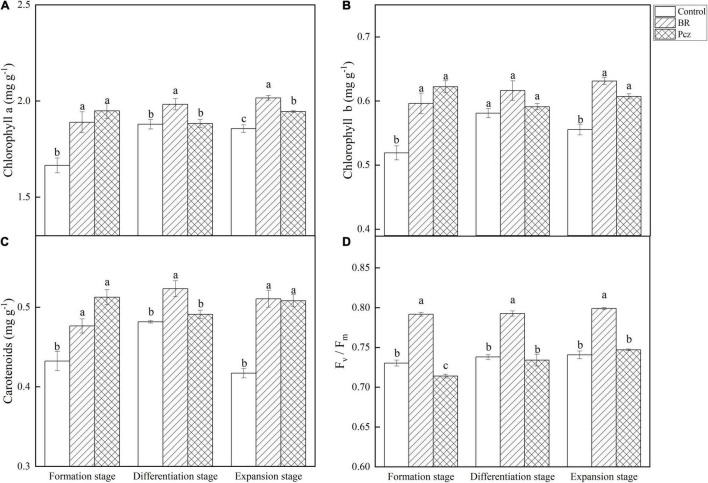
Chlorophyll a **(A)**, Chlorophyll b **(B)**, Carotenoids **(C)**, and F_*v*_/F_*m*_ **(D)** value in bulbil of *P. ternata* under BR and Pcz treatments. The bars with different letters are significantly different from each treatment (*p* < 0.05). Values are means of four replicates ± SE.

The chlorophyll a content was raised by 13.49, 5.54, and 8.56% under BR treatment at formation, differentiation, and expansion stages, and was increased by 17.02 and 4.76% under Pcz treatment at formation and expansion stages, compared to the control, respectively (Figure 4A). BR and Pcz treatments increased chlorophyll b content by 14.88 and 19.92% at the formation stage and 13.73 and 9.40% at the expansion stage, compared to the control, respectively (Figure 4B). The carotenoids content was increased by 10.29, 8.56, and 22.44% under BR treatment at formation, differentiation, and expansion stages, compared to the control, respectively (Figure 4C). Pcz treatment enhanced the carotenoids content by 18.66 and 21.87% at formation and expansion stages, compared to the control, respectively. The F_*v*_/F_*m*_ value was increased by 8.40, 7.41, and 7.88% under BR treatment at formation, differentiation, and expansion stages, compared to the control, respectively (Figure 4D). Pcz treatment significantly decreased the F_*v*_/F_*m*_ value at the formation stage compared to the control.

**FIGURE 5:**
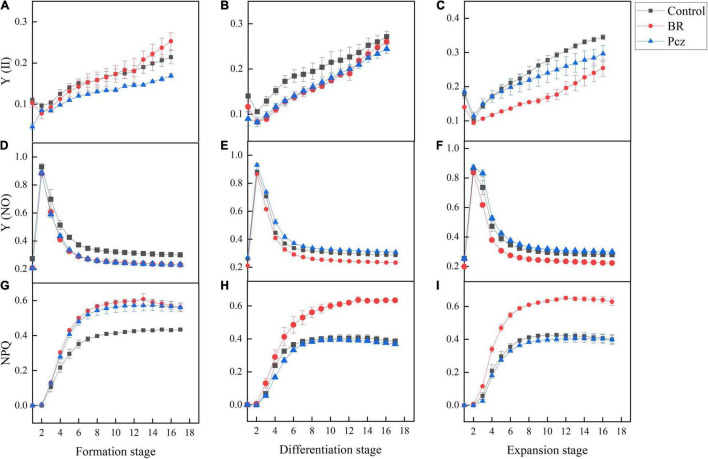
Chlorophyll fluorescence kinetic curves of *P. ternata* under BR and PCZ treatment. **(A–C)** Time-response of Y (II) at formation stage, differentiation stage, and expansion stage. **(D–F)** Time-response of Y (NO) at formation stage, differentiation stage, and expansion stage. **(G–I)** Time-response of NPQ at formation stage, differentiation stage, and expansion stage. Values are means of four replicates ± SE.

**FIGURE 6:**
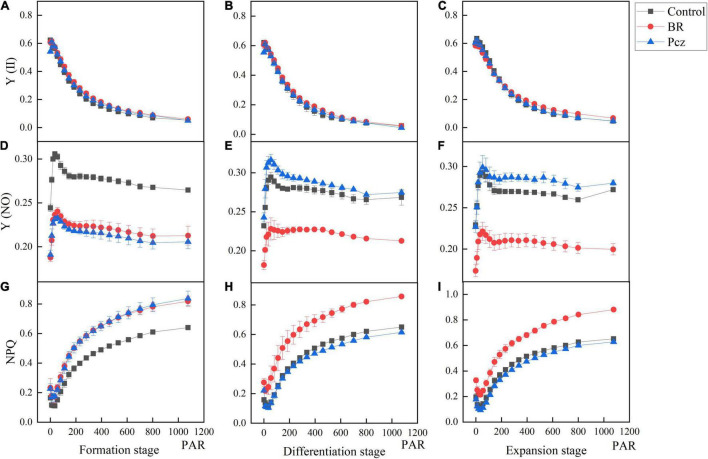
Chlorophyll fluorescence light response curve of *P. ternata* under BR and PCZ treatment. **(A–C)** Light-response of Y (II) at formation stage, differentiation stage, and expansion stage. **(D–F)** Light-response of Y (NO) at formation stage, differentiation stage, and expansion stage. **(G–I)** Light-response of NPQ at formation stage, differentiation stage, and expansion stage. Values are means of four replicates ± SE.

The effects of BR and Pcz treatments on the chlorophyll fluorescence kinetics curve were shown in Figure 5. The Y (II) value of Pcz treatment was lower than that of control and BR treatments at the formation stage. At the differentiation and expansion stages, the control had a higher Y (II) value compared to BR and Pcz treatments. The Y (NO) value of control group was higher than that of BR and Pcz treatments at the formation stage, and the BR treatment had a lower Y (NO) value compared to control and Pcz treatments at the differentiation and expansion stages. Compared to the control and Pcz treatments, the NPQ value was higher in BR treatment at the formation, differentiation, and expansion stages, which was more marked at the differentiation and expansion stages. The effects of BR and Pcz treatments on the chlorophyll fluorescence light response curve were shown in Figure 6. BR and Pcz treatments had no significant effects on the Y (II) value. At the formation stages, the control group had a higher Y (NO) value and had a lower NPQ value, compared to the BR and Pcz treatments, respectively. The Y (NO) value of BR treatment was lower than that of control and Pcz treatments, and the NPQ value of BR treatment was higher than that of control and Pcz treatments.

**FIGURE 7:**
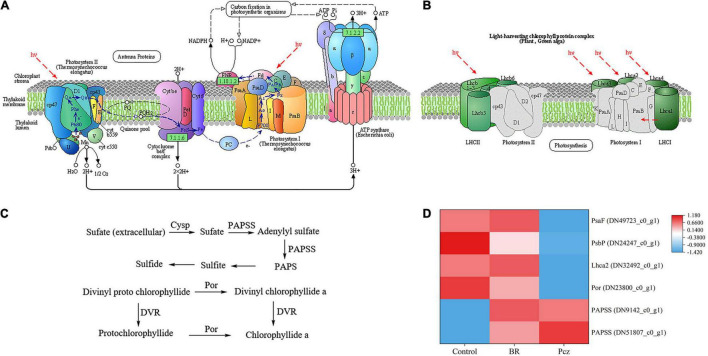
Analysis of differentially expressed levels related to photosynthesis pathway of *P. ternata* under BR and Pcz treatments. **(A)** The photosynthesis pathway. **(B)** The photosynthesis of antenna proteins. **(C)** Sulfur assimilation and chlorophyll a synthesis pathway. **(D)** The expression levels of genes related to the photosynthesis pathway. Blue indicates a lower expression level, whereas red indicates a higher expression level. All data showed the average mean of three biological replicates.

As shown in Figure 7, Pcz treatment evidently decreased the expression levels of photosystem I subunit III (PsaF), photosystem II oxygen-evolving enhancer protein 2 (PsbP), light-harvesting complex I chlorophyll a/b binding protein 2 (Lhca2), and protochlorophyllide reductase (Por) genes, and markedly increased the expression levels of two 3’-phosphoadenosine 5’-phosphosulfate synthase (PAPSS) genes, compared to the control, respectively. However, the expression level of PAPSS gene were enhanced by BR treatments, and there was no significant difference, compared to the control.

### Effects of Brassinolide Treatments on Endogenous Hormone

**FIGURE 8:**
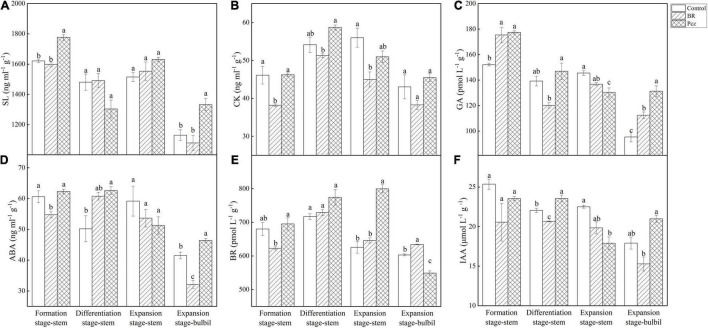
The endogenous SL **(A)**, CK **(B)**, GA **(C)**, ABA **(D)**, BR **(E)**, and IAA **(F)** levels in bulbil of *P. ternata* under BR and Pcz treatments. The bars with different letters are significantly different from each treatment (*p* < 0.05). Values are means of four replicates ± SE.

Pcz treatment increased the SL content in stem by 9.58% at the formation stage and in bulbil by 17.87% at the expansion stage, compared to the control, respectively (Figure 8A). The CK content in the stem was decreased by 17.17 and 19.66% under BR treatment at formation and differentiation stages, compared to the control, respectively (Figure 8B). The GA content in bulbil was raised by 17.91 and 37.68% under BR and Pcz treatments at the expansion stage, compared to the control, respectively (Figure 8C). The ABA content in bulbil was decreased by 22.54% under BR treatment and was enhanced by 11.77% under Pcz treatment, compared to the control, respectively (Figure 8D). BR treatment decreased the BR content in stem by 8.43% at the formation stage, and increased the BR content in bulbil by 5.13% at the expansion stage, compared to the control, respectively (Figure 8E). However, Pcz treatment raised the BR content in stem by 22.84% at the expansion stage, and decreased the BR content by 8.92% in bulbil at the expansion stage, compared to the control, respectively. The IAA content in the stem was decreased by 6.31% under BR treatment and was increased by 6.78% under Pcz treatment at the differentiation stage, compared to the control, respectively (Figure 8F). Pcz treatment decreased the IAA content in the stem by 20.47% at the expansion stage compared to the control.

**FIGURE 9:**
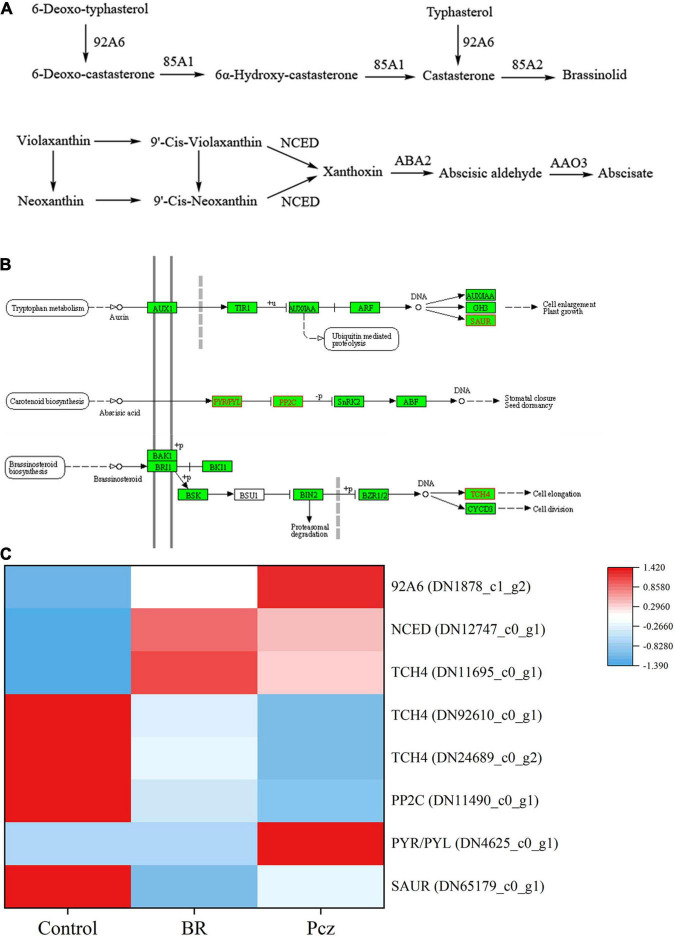
Analysis of differentially expressed levels related to hormone synthesis and signaling pathway in bulbil of *P. ternata* under BR and Pcz treatments. **(A)** The hormone synthesis pathway. **(B)** The hormone signaling pathway. **(C)** The expression levels of genes related to the hormone synthesis and signaling pathway. Blue indicates a lower expression level, whereas red indicates a higher expression level. All data showed the average mean of three biological replicates.

The function annotation of six databases indicated that eight DEGs were involved in the hormone signaling pathway in bulbil of *P. ternata* (Figure 9). The expression levels of 9-cis-epoxycarotenoid dioxygenase (NCED), typhasterol/6-deoxotyphasterol-2-alpha-hydroxylase (92A6), and xyloglucan: xyloglucosyl transferase TCH4 (TCH4) (DN11695_c0_g1) genes were significantly up-regulated in BR treatment, compared to the control, respectively. The expression level of auxin-responsive protein SAUR71 (SAUR) gene was significantly down-regulated in BR treatment, compared to the control, respectively. Pcz treatment markedly enhanced the expression levels of 92A6 and abscisic acid receptor PYR/PYL family (PYL) genes, and markedly decreased the expression levels of TCH4 (DN92610_c0_g1 and DN24689_c0_g2) and protein phosphatase 2C (PP2C) genes, compared to the control, respectively.

**FIGURE 10:**
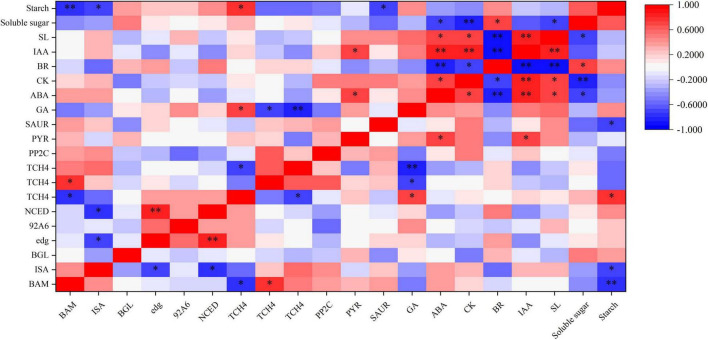
Spearman’s correlation coefficient between the starch and sucrose metabolism and hormone synthesis and signaling pathway in bulbil of *P. ternata* under BR and Pcz treatments. * and ^**^ indicated the significant correlations at the 0.05 and 0.01 level, respectively.

To better understand the relationship between hormone synthesis and signaling pathway and starch and sucrose metabolism pathway under BR and Pcz treatments, spearman’s correlation analysis was performed (Figure 10). The starch content was significantly negatively correlated with the expression levels of BAM, ISA, and SAUR genes and was significantly positively correlated with one TCH4 gene. The expression level of NCED gene was significantly positively correlated with the expression level of edg gene and was significantly negatively correlated with the expression levels of ISA gene. In addition, there were significant correlations between different endogenous hormone contents.

### qRT-PCR Validation of the Sequencing Data

qRT-PCR was used to estimate the expression patterns of DEGs identified by RNA-seq in starch and sucrose metabolism, photosynthesis pathway, and hormone signaling pathway. The relative expression levels of control vs. BR and control vs. Pcz under sequencing and qRT-PCR were compared (Supplementary Figure 3). The results showed that relative expression levels of RNA-seq were similar to that of qRT-PCR (*R*^2^ = 0.8036, Pearson’s correlation = 0.8964), which confirmed the reliability of sequencing data (Supplementary Figure 4).

## Discussion

Bulbils are important vegetative propagation organs in *P. ternata*. Our previous study showed that BR was involved in bulbil formation and development (Guo et al., 2021a,b). However, it is not clear about the molecular mechanism of BR regulating bulbil formation and development. Therefore, in order to further investigate the molecular information on the formation and development of *P. ternata* bulbil, we provided a transcriptomic dataset of *P. ternata*. These datasets are DEGs for the starch and sucrose metabolism, hormone synthesis and signal, and photosynthetic pathways under BR and Pcz treatments.

Starch and sucrose metabolism is important for bulbil formation and development. Interestingly, through histological observations, we found that the number and size of starch granules were increased by BR treatment compared to the control (Figure 1). One possibility is that BR was involved in the bulbil formation and development of *P. ternata* and promoted starch accumulation in bulbil of *P. ternata*. Therefore, we further explored the effect of BR treatment on starch content and starch and sucrose metabolism in bulbil of *P. ternata*. Our results showed that the tuber and bulbil yield, starch, and soluble sugar content were significantly enhanced by BR treatment, and the bulbil yield was significantly decreased by Pcz treatment (Figure 2). Studies with similar results showed that the soluble sugar content in the leaf of *Robinia pseudoacacia* was significantly higher in the 0.1 mg/L BR treatment under water stress compared to the control (Li et al., 2007). According to the KEGG, the metabolism of starch and sucrose in the bulbil of *P. ternata* was closely related to the activity of enzymes sucrose synthase (SUS), ectonucleotide pyrophosphatase/phosphodiesterase family member 1/3 (ENPP), glucose-1-phosphate adenylyltransferase (glgc), starch synthase (glgA), granule-bound starch synthase (WAXY), 1,4-alpha-glucan branching enzyme (GBE1), BAM, sucrase-isomaltase (SI), maltase-glucoamylase (MGAM), ISA, cellulose synthase (bcsA), edg, and BGL (Figure 3A). BR treatment significantly raised the expression levels of BGL and edg genes and decreased the expression levels of BAM and IAS genes (Figure 3B). The BAM activity was highly correlated with starch degradation during banana ripening, and the increased BAM activity was mainly associated with up-regulation of gene expression (do Nascimento et al., 2006). Li et al. (2020) reported that 0.05 mg/L BR treatment significantly increased the transcript level of BAM in guard cells, this may be related to different cellular functions, and BR treatment promoted starch degradation in guard cells, thus inducing stomatal opening. Silencing of the starch branching enzyme gene increased the amylose content in rice, which was associated with reduced ISA enzyme activity (Jiang et al., 2013). Chourasia et al. (2008) reported that the expression level of edg gene correlated with edg activity during mango ripening, which reduced cellulose/hemicellulose content. Overexpression of BGL gene increased BGL activity and promoted the fruit ripening process in grapes (Jia et al., 2016). These results indicated that BR treatment reduced starch catabolism to maltodextrin and maltose by decreasing BAM and ISA genes expression and increased cellulose catabolism to D-glucose by enhancing edg and BGL genes expression. The increases of starch and soluble sugar content in bulbil may be due to the reduced starch catabolism and increased cellulose catabolism, respectively. Studies with similar results showed that BR-induced increased cell wall glucose in switchgrass cell suspension cultures (Rao et al., 2017). BR promoted persimmon softening by regulating the expression of genes associated with cell wall degradation (He et al., 2018).

Sucrose is one of the products of photosynthesis, also plays an important role in the formation and development of bulbil. Plant photosynthetic processes depend on chlorophyll content, photosynthetic parameters, and chlorophyll fluorescence parameters (Stirbet and Govindjee, 2011). Yang A.J. et al. (2018) reported that BR treatment enhanced the photosynthetic pigments content and Fv/Fm value in *Leymus chinensis* grown under natural light. This result is in agreement with our finding that BR treatment significantly increased the chlorophyll a, chlorophyll b, and carotenoids content and Fv/Fm value (Figure 4). In addition, Pcz treatment also increased the content of photosynthetic pigments in *P. ternata*. Similar result was observed that Pcz treatment increased the chlorophyll content of maize plants under drought stress (Rajasekar et al., 2015). Fv/Fm and Y (II) reflect the potential maximum photosynthetic capacity and actual photochemical efficiency of photosystem II (PSII), respectively. On the fluorescence kinetic curve, BR treatment reduced the PS II value at the formation and expansion stages (Figure 5). However, on the light response curve, BR treatment had no effect on the PSII value (Figure 6). Similar results showed that BR treatment significantly decreased Y (II) value in small black bean sprouts stored for 5 days at 4°C (Xue et al., 2021). Y (NO) is an important indicator of photodamage in PSII, indicating the passive dissipation of energy in the form of heat and fluorescence values (Yuan et al., 2017). NPQ indicates the ability to dissipate excess light energy as heat by non-photochemical quenching and is an important indicator of photoprotection in PSII (Xue et al., 2021). In this paper, on the fluorescence kinetic and light response, BR treatment reduced the Y (NO) value and enhanced the NPQ value at the formation, differentiation, and expansion stage. These results suggested that BR treatment improved the photoprotection ability of *P. ternata* by increasing the dissipation of excess light energy to heat, thus reduced the photodamage in the PSII center. Zhang et al. (2019) study showed that BR treatment protected chloroplast structure under cold stress in tungsten seedlings, thus maintaining normal chloroplast function. Based on the KEGG enrichment analysis, three DEGs were found to be annotated in the photosynthesis pathways, and these DEGs were significantly down-regulated by Pcz treatment (Figure 7). The PsaF subunit of PSI had unnecessary auxiliary roles in the function and organization of the complex (Xu et al., 1994). PsbP protein is an exogenous subunit of PSII and is involved in photosynthetic water oxidation. PsbP deficiency inhibited the normal development of plants and reduced the PSII quantum yield (Ifuku et al., 2005). These findings confirmed that BR plays an important role in photosynthesis of *P. ternata*. In addition, Pcz treatment significantly increased the expression levels of PAPSS, indicating the promoting roles of sulfur assimilation by Pcz treatment. In most photosynthetic organisms, Por plays a key role in light-independent (bacterial) chlorophyll biosynthesis (Nomata et al., 2005). However, Pcz treatment significantly reduced the expression level of Por, suggesting that Pcz treatment decreased chlorophyll synthesis in bulbil of *P. ternata*.

Furthermore, the correlation analysis between starch and sucrose metabolism and hormone signal pathway showed that BR interacted with other hormones to regulate starch and sucrose metabolism pathway (Figure 10). SL is a class of terpene phytohormones that regulates branching, germination, and mesocotyl length in plants. The SL content in bulbil of *P. ternata* was significantly increased by Pcz treatment. This is in line with previous results that GA biosynthesis inhibitor (1 μM uniconazole or 1 μM Paclobutrazol) increased the SL content in the root culture of rice (Ito et al., 2017). BR treatment reduced the endogenous CK content and increased the endogenous BR content in bulbil of *P. ternata*. This reason may be that BR and CK play similar roles in cell division, and BR can be used as a substitute for CK in callus and suspension cultures of *Arabidopsis* (Hu et al., 2000). BR treatment increased the endogenous BR content in bulbil of *P. ternata*, this may be related to that BR treatment enhanced the BR biosynthesis in bulbil of *P. ternata* by increasing the expression level of 92A6 genes. In addition, Pcz treatment significantly decreased the BR content in bulbil of *P. ternata*, and significantly enhanced the BR content in the stem of *P. ternata* at the expansion stage. However, Pcz treatment significantly increased the expression level of 92A6 genes, indicating that the increased BR content in the stem of *P. ternata* at the expansion stage may be related to that Pcz treatment promoted the BR biosynthesis pathway in bulbil. A similar result was reported by Chmur and Bajguz (2021), who found that 0.01–1.00 μM BR treatments significantly enhanced the BR content in *Wolffia arrhizal*, and BR inhibitors (brassinazole) treatment decreased the endogenous level of BR. The expression level of one TCH4 gene was increased by BR treatment and was positively correlated with starch content in bulbil. These results indicated that BR treatment promoted starch accumulation by enhancing the expression level of the TCH4 gene in the BR signal transduction pathway. Nakamura et al. (2003) study showed that the IAA level in *Arabidopsis* seedlings did not change after BR treatment. This result is in agreement with our finding that BR and Pcz treatments had no significant effect on the endogenous IAA content in bulbil of *P. ternata*. However, BR treatment significantly decreased the expression level of SAUR and slightly reduced the endogenous IAA content in bulbil of *P. ternata*, which may reduce the signal transduction of IAA in bulbil. Moreover, the expression level of SAUR gene was negatively correlated with starch content in bulbil. Previous studies showed that IAA inhibited bulbil development in *Agave tequilana* (Abraham Juarez et al., 2015). These results suggested that BR treatment promoted bulbil development in *P. ternata* by down-regulating the expression level of the SAUR gene in the signal transduction pathway of IAA. The endogenous ABA level in bulbil of *P. ternata* was significantly decreased by BR treatment and was significantly increased by Pcz treatment. A similar result was reported by Ha et al. (2018), who found that BR treatment reduced ABA content in *Arabidopsis* by down-regulating the expression level of ABA biosynthetic genes. In cereals, ABA content was negatively correlated with starch synthesis (Zhu et al., 2011). However, the expression level of NCED gene was negatively correlated with the expression level of ISA gene under BR and Pcz treatments. One possible reason is that BR treatment decreased the ABA content in bulbil of *P. ternata* and increased the expression level of the ABA biosynthetic gene NCED through a negative feedback regulatory mechanism. Moreover, the expression level of NCED was positively correlated with the expression level of edg genes. Sun et al., (2012) also reported that the transcription level of genes encoding major cell wall catabolic enzymes was down-regulated with decrease NCED gene expression and activity. The expression levels of PYR/PYL and PP2C genes were up-regulated by Pcz treatment, indicating that Pcz treatment enhanced the signal transduction of ABA. The bulbil formation and development were inhibited by IAA, GA, and SL (Abraham Juarez et al., 2015; Navarro et al., 2015). Pcz treatment increased the level of GA, SL, ABA, and IAA in bulbil of *P. ternata*, which may inhibit the formation and development of *P. ternata* bulbil.

## Conclusion

BR treatment promoted bulbil formation and development of *P. ternata* by regulating the starch and sucrose metabolism, photosynthesis pathway, and hormone signaling pathway. The number and size of starch granules were increased by BR treatment and the starch and soluble sugar content were increased by 12.87 and 19.32% under BR treatment. BR treatment reduced the starch catabolism to maltodextrin and maltose, and increased the cellulose catabolism to D-glucose by regulating the expression level of BAM, ISA, edg, and BGL genes. The photoprotective capacity of *P. ternata* was enhanced by BR treatment, which reduced the photodamage in PSII centers by increasing the dissipation of excess light energy to heat. The endogenous BR biosynthesis of *P. ternata* was inhibited by Pcz treatment, which reduced the function of PS I complex and photosynthetic water oxidation of PS II by reducing the expression of PsaF and PsbP genes in bulbil of *P. ternata*, respectively. BR regulated the bulbil formation and development by regulating endogenous hormones levels, such as SL, CK, GA, ABA, BR, and IAA in *P. ternata*. BR treatment enhanced the BR biosynthesis in bulbil of *P. ternata* by increasing the expression level of 92A6 genes. The signal transduction of BR was promoted under BR treatment by enhancing the expression level of the TCH4 gene and was inhabited under Pcz treatment by decreasing the expression level of the TCH4 gene. In addition, BR treatment reduced the signal transduction of IAA in bulbil of *P. ternata* by decreasing the expression level of the SAUR gene. Pcz treatment promoted the signal transduction of ABA by increasing the expression level of the PYR/PYL gene and decreasing the expression level of PP2C gene.

## Data Availability Statement

The original contributions presented in the study are publicly available. This data can be found here: National Center for Biotechnology Information (NCBI) BioProject database under accession number PRJNA719943.

## Author Contributions

CG, JC, and XY designed the experiments and contributed to writing and revising the manuscript. CG, JL, ML, XX, and YC performed the experiments. CG analyzed the data. JL, JC, and XY supervised the study. All authors contributed to the article and approved the submitted version.

## Conflict of Interest

The authors declare that the research was conducted in the absence of any commercial or financial relationships that could be construed as a potential conflict of interest.

## Publisher’s Note

All claims expressed in this article are solely those of the authors and do not necessarily represent those of their affiliated organizations, or those of the publisher, the editors and the reviewers. Any product that may be evaluated in this article, or claim that may be made by its manufacturer, is not guaranteed or endorsed by the publisher.

## Supplementary Material

Supplementary Figure 1 Venn diagram of DEGs in control vs. BR, control vs. Pcz, and BR vs. Pcz groups.

Supplementary Figure 2 Cluster analysis of DEGs under control, BR, and Pcz treatments.

Supplementary Figure 3 Comparison of the expression patterns of genes involved in the starch and sucrose metabolism, photosynthesis pathway, and hormone signaling pathway in bulbil of *P. ternata* under control, BR, and Pcz treatments by qRT-PCR and RNA-seq. values are means of three replicates ± SE.

Supplementary Figure 4 Correlation plot between RNA-seq and qRT-PCR for the relative expression of unigenes.

Supplementary Table 1 Primers used for quantitative real-time PCR.

Supplementary Table 2 Length distribution of assembled unigenes.
