# Supplementary material for: Regulation Mechanism of Exogenous Brassinolide on Bulbil Formation and Development in Pinellia ternata
Source: Front Plant Sci. 2022 Jan 5;12:809769. doi: 10.3389/fpls.2021.809769 (PMC8766408; doi:10.3389/fpls.2021.809769)
Supplement: Supplementary Figure 1 — Venn diagram of DEGs in control vs. BR, control vs. Pcz, and BR vs. Pcz groups. [file Data_Sheet_1.docx]

Supplementary Table 1 Primers used for quantitative real-time PCR

| Gene name | Forward (5'-3') | Rearward (5'-3') |
| --- | --- | --- |
| PAPSS | TCCTTCCATTTAGGGTTGCTG | AAATCTTGTGGTCTCGACGG |
| Por | CCCTTGGTGATGTACTTCTGG | AACACGCAGCTCTTCAGG |
| Lhca2 | TTCCGGGAGACTTTGGTTTC | ACTGCACGTTCCATCTCAAG |
| PsbP | GAGGTGGGTTGGATCAAGAC | AGCTGAACATACCGTCCAAG |
| PsaF | GCCTACAGAGAACTCGTCAC | CCACCCTTCTCCTTCCAATG |
| auxin | TTAGATCCAAGCCACCTGTTAG | ACCTTGTTCTCATCTAGTTTGCC |
| gibberellin | TGGCTTCTCCTTGCTCTTC | CATATCTTGTACCTCGACGCC |
| ABA | GAATCTGGAAAAGGCGTTGC | CTCAACAGTTCAACCGACAAC |
| 92A6 | CCTACTTGGGCCGTTTATCTAG | ATGAGGTTGAGATTGCCGAC |
| cytokinin | CACAGTGATCCCCAGGTTTAC | TCATCGACCTCTTGCTGTTG |
| NCED | AGAGATGACGCCGATTCTTG | TTTCTCTGTTCCACGACTTCG |
| SAUR | GGAAGGTGATCAAGCAGGTG | TTCTTTGCTCCCCACGAC |
| PYR/PYL | GGAGGGTCACGAAATCACAC | CCCGTGCATGAATCCTAGC |
| PP2C | CCATCGTTTTCCTTCAGTGC | ACAAGTGGTTCTCGCAAGG |
| MAPKK17-18 | GCCGCATTACACGAAACAATAG | ACGCTTCCAGTTCGAGATTC |
| ERF1 | TGGAGAGTGTGGTAGTGGAG | TTAACTAGGGCCACGTCATG |
| ChiB | GTGGTTTCATGGGAGGTCTG | CCAGCAACTTCTACACCTACG |
| TCH4 | CTGCCGTACACCCTCATC | TCCACACCTACTCCATCCTC |
| PR1 | ATCGTGGTACTGTGCGTG | CGGTTGGCATAGTTCTGGG |
| CaM4 | CCAATATCGGGTCCATGATCG | GTCCATCACCCTGAACACC |
| edg | GCATCGACAAGAACACCATC | TCCAAAACTCCCGATCAACAC |
| BGL | GTAAGATCCGTAGAAGTAGCCG | GAGCCCACAGATACAGCAAG |
| ISA | GACATGGCTATATCATTTTCCTGG | GGAGCAGAAACTAAATGTGACATAC |
| BAM | CATCGCCATTTGATATTCCGG | TCGTAGCACTAAAACTCGCC |

Supplementary Table 2 Length distribution of assembled unigenes

| Length | Number of Transcript | Percent of Transcript |
| --- | --- | --- |
| 200~500 | 107517 | 50% |
| 501~1000 | 53508 | 25% |
| 1001~1500 | 21759 | 10% |
| 1501~2000 | 12463 | 6% |
| 2001~2500 | 7335 | 3% |
| 2501~3000 | 4315 | 2% |
| 3001~3500 | 2434 | 1% |
| 3501~4000 | 1548 | 1% |
| 4001~4500 | 897 | 0% |
| >4500 | 1808 | 1% |





Supplementary Fig. 1 Venn diagram of DEGs in control vs BR, control vs Pcz, and BR vs Pcz groups.


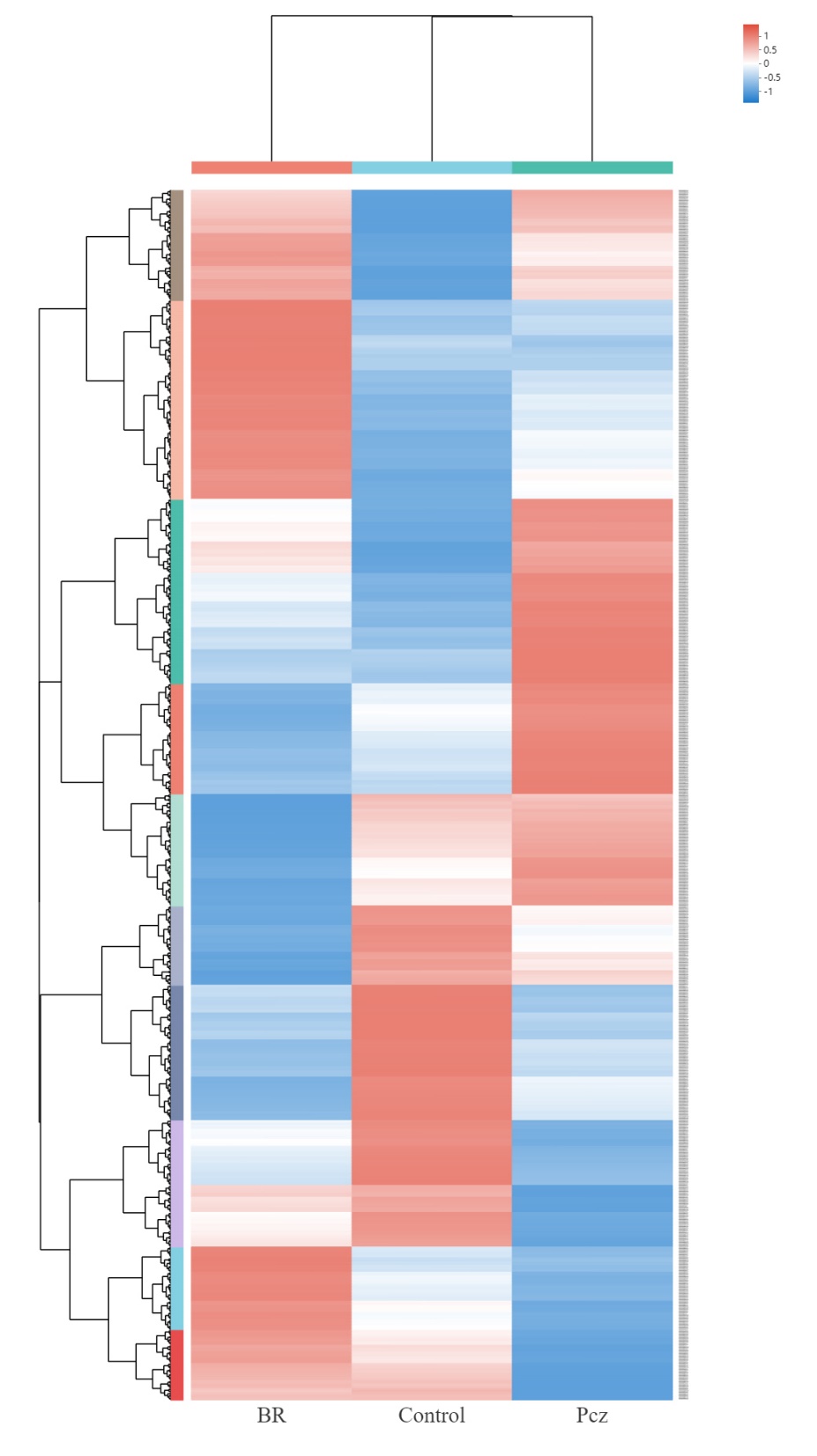


Supplementary Fig. 2 Cluster analysis of DEGs under control, BR, and Pcz treatments.





Supplementary Fig. 3 Comparison of the expression patterns of genes involved in the starch and sucrose metabolism, photosynthesis pathway, and hormone signaling pathway in bulbil of *P. ternata* under control, BR, and Pcz treatments by qRT-PCR and RNA-seq. values are means of three replicates ± SE.





Supplementary Fig. 4 Correlation plot between RNA-seq and qRT-PCR for the relative expression of unigenes.
